# Investigations of Curcumin and Resveratrol on Neurite Outgrowth: Perspectives on Spinal Muscular Atrophy

**DOI:** 10.1155/2014/709108

**Published:** 2014-07-02

**Authors:** Gamze Bora-Tatar, Hayat Erdem-Yurter

**Affiliations:** Department of Medical Biology, Faculty of Medicine, Hacettepe University, Sıhhiye, 06100 Ankara, Turkey

## Abstract

Spinal Muscular Atrophy (SMA) is an autosomal recessive neurodegenerative disease with progressive muscle weakness and atrophy. SMA is caused by low levels of the Survival of Motor Neuron (SMN) protein, which also leads to neurite outgrowth defects in neuronal cells. Rescue of the outgrowth defect is thought to be a strategy for SMA treatment. Polyphenolic histone deacetylase (HDAC) inhibitors might be good candidates due to their neuritogenic properties. In the present study, it was investigated whether neurite outgrowth defects could be rescued by curcumin and resveratrol, which are SMN-inducing polyphenols, having HDAC inhibition activity. According to our results, although curcumin and resveratrol failed to restore the neurite outgrowth defects, the SMN protein was found to be necessary for the neurite-promoting activity of curcumin in neuron-like PC12 cells.

## 1. Introduction

The nervous system harbors functionally diverse neurons that possess a similar cellular morphology. Neurons are characterized by multiple protrusions called neurites, and neurites are important for polarity through their differentiation into axons and dendrites [1–3].

Correct establishment of this structure is crucial and abnormalities in these structures have been shown in several neurodegenerative diseases, including Spinal Muscular Atrophy (SMA) [4, 5]. SMA is an autosomal recessive disease that is characterized by the progressive loss of spinal cord alpha motor neurons. Neurodegeneration causes proximal muscle weakness and atrophy, which affects motor skills and, together with the age of onset, defines the types of SMA (I–IV) [6, 7].

The main cause of SMA is the homozygous deletion of exons 7 and 8 of the Survival of Motor Neuron 1 (*SMN1*) gene [8, 9]. A nearly identical copy,* SMN2*, presents only in humans, which essentially differs from* SMN1* by a single nucleotide change in exon 7. This variation disrupts correct splicing of SMN2 mRNA and leads to the reduction in the amount of the full-length protein. This amount is not sufficient to prevent SMA; however, multiple* SMN2* copies directly impact clinical severity [7, 10].

SMA emerges due to low levels of the SMN protein, which is found in the cytoplasm, nuclear bodies (gems and Cajal bodies), neurites, and also the growth cone [11–13]. The SMN functions as an assembly protein for small nuclear ribonucleoprotein particles involved in pre-mRNA splicing. In addition to its housekeeping function, the SMN plays a role in actin dynamics, axonal transport, and neurite outgrowth in neurons [12, 14]. Previous studies showed that SMN deficiency leads to axon/neurite outgrowth defects. Truncated motor axons were reported in the SMN knockdown zebrafish model* in vivo*. Similarly, primary motor neuron culture from SMA mouse embryos showed reduced axon growth* ex vivo* [15–17].* In vitro* studies also showed that, in the rat PC12 cell line, knockdown of SMN protein resulted in shorter neurites [18, 19]. A reduction in neurite length was also demonstrated in induced pluripotent stem cell-derived motor neuron culture, which was established from SMA patient fibroblast cells, and ectopic SMN expression restored these defects [20].

Hence, rescue of defective neurite outgrowth could be a reasonable therapeutic strategy for SMA. Polyphenolic compounds and histone deacetylase (HDAC) inhibitors may be good candidates for this purpose due to their neuroprotective properties [21, 22]. Many signaling pathways that play a role in oxidative stress, inflammation, and neuronal differentiation were reported as targets of these compounds, demonstrating their usefulness in multisystem disorders like SMA [23–26]. Curcumin and resveratrol are compounds that possess both HDAC inhibitory and neuroprotective properties [27, 28]. Curcumin, which is extracted from the rhizomes of* Curcuma longa*, is the main ingredient of the Indian spice turmeric and resveratrol (*trans*-*3,4* ′*,5-trihydroxystilbene*) is a grape polyphenol, mostly found in red wine. Both compounds have a neurite-promoting effect, which was shown in several reports [29–31]. The effects of curcumin and resveratrol on a cell culture model for SMA were also reported, and it was shown that both increased full-length* SMN2* gene expression in a SMA patient fibroblast cell line [32–34]. Considering these findings, in the present study, we aimed to restore the neurite outgrowth defect observed in SMA, using curcumin and resveratrol. A well-known* in vitro* model for differentiation studies derived from rat pheochromocytoma, the PC12 cell line, is used to investigate the potencies of polyphenols on neurite outgrowth.

## 2. Materials and Methods

### 2.1. Cell Culture and Treatments

PC12 and SMN knockdown (85%) stable PC12 cell lines were kindly provided by Dr. Rashmi Kothary (Ottawa Hospital Research Institute, Ottawa, ON, Canada). The cells were cultured on rat tail collagen type I (Cultrex) coated plates. The PC12 cells were grown in Dulbecco's modified eagle medium (DMEM) with 4.5% glucose (Biochrom), supplemented with 10% horse serum (Invitrogen), 5% fetal bovine serum (FBS) (Biochrom), 1% antibiotic/antimycotic solution (Invitrogen), 1% nonessential amino acids (Invitrogen), and 1% L-glutamine (Biochrom). For the neurite outgrowth experiments, the cells were differentiated in DMEM with 1% FBS, 1% antibiotic/antimycotic solution, 1% nonessential amino acids, 1% L-glutamine, and 100 ng/mL nerve growth factor (NGF) 2.5S (Millipore, Chemicon). The SMN knockdown PC12 cells were cultivated in complete media, including 1 mg/mL G418 (Invitrogen).

For the neurite outgrowth experiments, wild-type (wt) and SMN knockdown PC12 cells were transferred into 6-well plates at a density of 21 × 10^3^ and 28 × 10^3^ cells/well, respectively. Curcumin (Sigma) and resveratrol (Sigma) were dissolved in DMSO (Applichem) immediately before each experiment and diluted to a final concentration in differentiation media prior to use (final DMSO concentration 0.1%). Wild-type cells were differentiated for 3 and 7 days in differentiation medium, with or without 500 nM curcumin and 5 *μ*M resveratrol, at 5% CO_2_ in an incubator at 37°C. The SMN knockdown cells were also treated with curcumin and resveratrol and differentiated for 3 days under the same conditions. Valproate (VPA, Sigma), one of the HDAC inhibitors, was used at a 2 mM concentration as a positive control and the cells were differentiated for 3 days. All of the treatments were performed in triplicate.

### 2.2. Neurite Outgrowth Analysis

At the end of the differentiation period, the cells were observed under an inverted light microscope (Leica DMIL) and pictures of the 3 fields in each well were taken. Morphometric analyses were performed using Qwin Image Analysis Software (Leica). The length of the longest neurite was measured per cell via drawing a straight line along the neurite, as previously described in [18]. Only neurites that were equal to or longer than twice the cell body diameter were included in the outgrowth analyses. Data from the 3 fields in each well were pooled and the lengths of over 150 neurites were included for statistical analysis. The neurite outgrowth experiments were performed in triplicate.

### 2.3. Cell Viability

To assess cell viability,* 4* ′*,6-diamidino-2-phenylindole* (DAPI) (Molecular Probes-Invitrogen, 1 : 1000 in phosphate buffered saline (PBS)) stainings were performed for 1 min at room temperature. Luminous nuclei were an indicator of nonviable cells and viability was shown as the percentage of live cells [35].

### 2.4. Immunocytochemistry

PC12 cells were differentiated on glass coverslips that were coated with both poly-L-lysine (Sigma) and mouse laminin (R&D Systems, Cultrex). After 3 days of differentiation, the cells were fixed in 100% ice-cold methanol for 5 min at −20°C and were blocked for 1 h with 1X PBS containing 0.1% Tween 20 (Sigma), 10% goat serum (Sigma), and 10% bovine serum albumin (Sigma). Mouse monoclonal anti-SMN primary antibody (1 : 100, BD Biosciences) and anti-mouse Alexa-fluor 488 labeled secondary antibody (1 : 500, Molecular Probes-Invitrogen) were both applied to the cells for 1 h at room temperature, and nuclear staining was performed using DAPI. After mounting in ProLong Gold Antifade solution (Molecular Probes-Invitrogen), the cells were visualized under a fluorescent microscope with an appropriate filter. Immunostainings were performed in triplicate. SMN-positive nuclear bodies were counted manually and the nuclear body numbers of the curcumin treated cells were compared to the untreated control samples. Data were calculated as the nuclear body number per 100 nuclei [11].

### 2.5. Immunoblotting

PC12 cells were differentiated with 500 nM curcumin for 3 and 7 days and were then collected in a buffer solution containing 10% sodium dodecyl sulfate (SDS) (Carlo Erba), 62.5 mM Tris-hydrochloride (Sigma), 5 mM EDTA, and a complete protease inhibitor cocktail tablet (Roche). The total protein was extracted using sonication (Sonics Vibracell) and centrifugation. The concentration of proteins was determined using a bicinchoninic acid assay (Pierce). Equal amounts of proteins (20 *μ*g) were loaded on SDS-polyacrylamide gels and were subsequently transferred onto nitrocellulose membranes using semidry blotting (Bio-Rad). The membranes were incubated with mouse monoclonal anti-SMN (1 : 2000, BD Biosciences) and rabbit polyclonal anti-actin (1 : 5000, Sigma) primary antibodies, and horseradish peroxidase conjugated anti-mouse (1 : 8000, Sigma) and anti-rabbit (1 : 2000, Amersham) secondary antibodies. ECL Plus (Amersham) was used according to the manufacturer's instructions to visualize the signals. The SMN levels of the curcumin treated samples were quantified according to the SMN/actin ratio and the untreated controls. The immunoblottings were performed in triplicate.

### 2.6. Statistical Analysis

Statistical analyses were performed using Graphpad Prism software (version: 6.04). Data were analyzed by two-way ANOVA with Bonferroni posttest, Mann-Whitney* U*, and Kruskal-Wallis tests. Results were considered significant at *P* < 0.05.

## 3. Results

### 3.1. Effects of Resveratrol and Curcumin on Neurite Outgrowth in Wild-Type PC12 Cells

**Figure 1:**
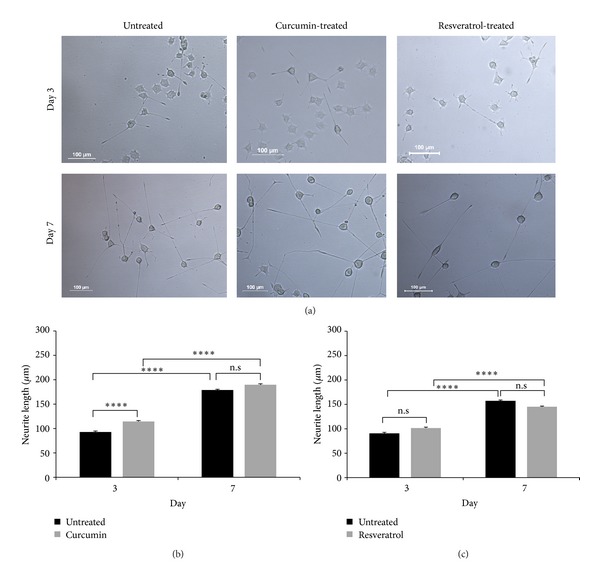
Neurite outgrowth analysis after curcumin and resveratrol treatments in wt PC12 cells. (a) Examples of the untreated, curcumin and resveratrol treated PC12 cells at 3 and 7 days of differentiation. Scale bar; 100 *μ*m (b) quantitative measurements of the neurite lengths at 3 and 7 days of differentiation after 500 nM curcumin treatment, two-way ANOVA (*P* = 0.22), two group comparisons with Mann-Whitney *U*-test (****P < 0.0001). (c) Neurite length measurements at 3 and 7 days of differentiation after 5 *μ*M resveratrol treatment, two-way ANOVA (*P* = 0.003) with Bonferroni posttest (****P < 0.0001). Data are presented as means ± standard error (SE), *n* = 3, n.s: non significant.

The influence of curcumin and resveratrol on cell death was measured at a nanomolar and micromolar range via DAPI staining. The optimum concentrations of curcumin and resveratrol for outgrowth studies were determined as 500 nM and 5 *μ*M, respectively, due to 99% viability of the cells. The PC12 cells were exposed to curcumin and resveratrol for 3 and 7 days, and it was shown that the neurites of curcumin treated cells were significantly longer at 3 days of differentiation compared to the untreated controls. Although it was not statistically significant, a slight increase in neurite length was also detected at 3 days of differentiation after resveratrol treatment. A significant change was not visible in the neurite lengths at 7 days of differentiation, with neither the curcumin nor resveratrol administration (Figure 1).

### 3.2. Effects of Resveratrol and Curcumin on SMN Knockdown PC12 Cells

**Figure 2:**
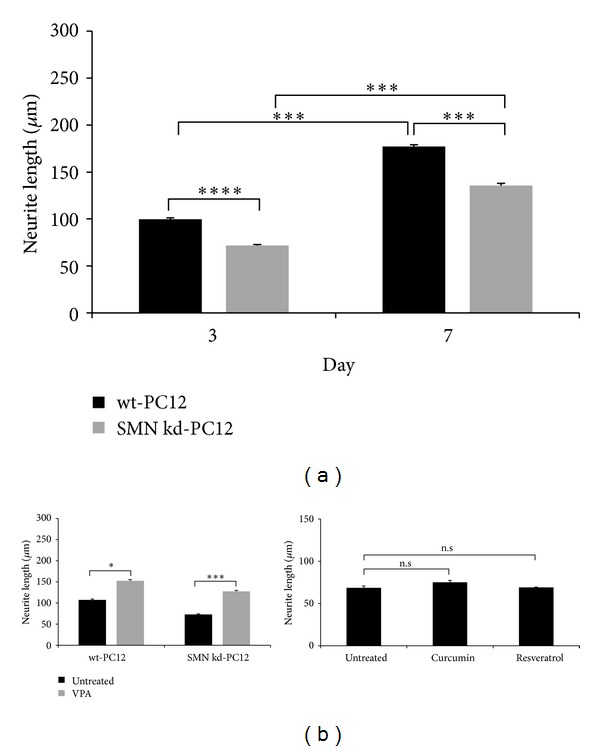
Neurite outgrowth analysis of the SMN knockdown PC12 cells and compound treatments. (a) Comparison of the neurite lengths between wt and SMN knockdown PC12 cells at 3 and 7 days of differentiation, two-way ANOVA (*P* = 0.09), two group comparisons with Mann-Whitney *U*-test (***P < 0.001, ****P < 0.0001). (b) VPA (2 mM) was applied to both the wt and SMN knockdown cells for 3 days as a positive control, two-way ANOVA (*P* = 0.33), two group comparisons with Mann-Whitney *U*-test (*P < 0.05, ***P < 0.001). Neurite lengths of the curcumin (500 nM) and resveratrol (5 *μ*M) treated SMN knockdown cells at 3 days of differentiation, Kruskal-Wallis test. Data are presented as means ± SE, *n* = 3, n.s: non significant.

SMN deficiency causes neurite outgrowth defects* in vitro* [16–18]. To show this defect, the neurite lengths of SMN knockdown PC12 cells were analyzed. It was found that the neurites of the knockdown cells were significantly shorter than those of the wild-type cells at both 3 and 7 days of differentiation (Figure 2(a)). VPA, a widely used HDAC inhibitor, was chosen as a positive control, and both the wild-type and SMN knockdown cells were differentiated for 3 days in 2 mM of VPA-containing medium. Our results showed that VPA significantly promotes neurite outgrowth and rescues the neurite outgrowth defect in SMN knockdown cells by increasing the neurite length about 54 *μ*m. (Figure 2(b)). To test whether curcumin and resveratrol can induce neurite elongation as well as VPA, SMN knockdown cells were treated with the indicated concentrations of curcumin and resveratrol. The neurite lengths of the compound treated cells could be measured only after 3 days of differentiation, because of the negative effects of the compounds on cell viability at 7 days. We showed that neither curcumin nor resveratrol could promote neurite elongation when compared to the untreated controls (Figure 2(b)).

### 3.3. Effects of Curcumin on SMN Protein Expression

**Figure 3:**
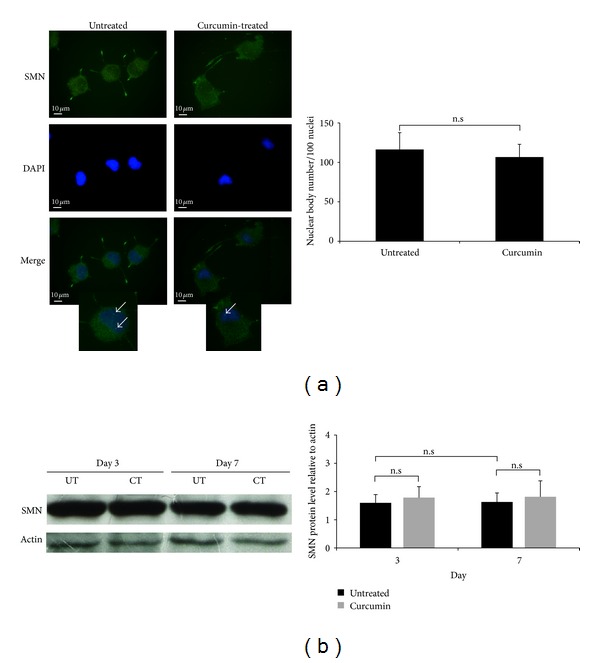
Effects of curcumin on SMN protein expression. (a) Representative images and quantification of the nuclear bodies of 3-day differentiated wt PC12 cells: SMN staining (green), DAPI staining (blue), and the merged images of untreated and curcumin treated cells. Arrows indicate the nuclear bodies. Scale bar; 10 *μ*m. Mann-Whitney *U*-test. (b) Western blot images and quantification of the total SMN protein level after 3 and 7 days of curcumin treatment in wt PC12 cells, two-way ANOVA (*P* = 0.99), two group comparisons with Mann-Whitney *U*-test. Data are presented as means ± SE. (UT, untreated; CT, curcumin treated, n.s: non significant, *n* = 3).

The neurite outgrowth analysis revealed that neurite promotion by the curcumin in the wild-type cells disappeared when the SMN was knocked down. Previous reports indicated that the SMN seems to be essential for the correct establishment of neurite morphology [18, 19]. Additionally, curcumin was reported as SMN-upregulating compound in SMA patient fibroblast cells [33, 34]. In light of these findings, we hypothesized that SMN protein upregulation might be a possible mechanism for neurite elongation. To test this hypothesis, nuclear and total SMN proteins were investigated only in the wild-type cells, because neurite elongation was not detected in the SMN-depleted cells. Since resveratrol was not able to induce neurite outgrowth effectively, protein studies were performed with curcumin and the nuclear SMN level was investigated by immunofluorescence staining of the nuclear bodies in the wild-type cells. No change was detected in the nuclear body number in response to the curcumin. The total SMN protein level was quantified after 3 and 7 days of curcumin treatment, and similar results were detected by Western blot. As a result of the experiments, we showed that curcumin increases neither the nuclear body number nor the total protein level at any differentiation period of the wild-type PC12 cells (Figure 3).

## 4. Discussion

SMA is a prevalent neurodegenerative disorder with a neurite outgrowth defect, which is caused by a SMN protein deficiency. Rescue of the defect may help to slow down the neurodegeneration process; thus, it may be included in therapeutic strategies that have been developed for SMA [36, 37]. Neuroprotective compounds, like polyphenols and HDAC inhibitors, may be used for restoring of the outgrowth defect. It has been shown that polyphenols like chlorogenic acid and (−)-epigallocatechin-3-gallate promote neurite outgrowth in hippocampal neuronal cells and PC12 cells, respectively [38, 39]. Several HDAC inhibitors, such as VPA, trichostatin A, and suberoylanilide hydroxamic acid, also promote the differentiation of adult hippocampal neuronal progenitors, embryonic neural stem cells, and subventricular zone precursor cells, respectively [40–43].

In the present study, we investigated whether polyphenols possessing HDAC inhibition activity, resveratrol, and curcumin can restore the neurite outgrowth defect. To analyze the effects of the compounds, we used the PC12 cell line, a widely used neuron-like cell culture model for neuronal differentiation studies [44]. According to our results, curcumin significantly increased the neurite lengths of the PC12 cells at 3 days of differentiation. At the same time period, a slight but not statistically significant increase was detected in response to resveratrol. The effects of resveratrol and curcumin on neurite morphology were demonstrated by several studies. It has been reported that curcumin repaired the distorted neurites around the senile plaques in an Alzheimer mouse model, increased the number of neurites of predifferentiated PC12 cells, and promoted neurite outgrowth without the presence of NGF [45–47]. Similarly, resveratrol also induced neurite elongation in primary cortical, hippocampal neurons, and also PC12 cells [48, 49]. In the present study, it was observed that curcumin has more prominent effect on neurite elongation than resveratrol. We previously screened the pan-HDAC inhibition activities of these compounds using HeLa nuclear extracts and determined that curcumin is a more powerful HDAC inhibitor than resveratrol. Hence, it is likely that curcumin is capable of enhancing the neurite elongation of either precursor or differentiated neurons by altering the histone acetylation pattern of differentiation-related genes [27, 50, 51].

Several studies reported that the absence of the SMN protein results in neurite outgrowth defects [16–19]. In our study, we used SMN knockdown PC12 cells, which were previously established as a stable cell line with the knockdown of 85% SMN protein [18]. To show the neurite outgrowth defect, we differentiated the SMN knockdown cells and showed that the neurites were significantly shorter than those of the wild-type cells during differentiation. In order to investigate whether resveratrol and curcumin promote neurite elongation in the SMN-depleted cells, they were both administered under the same experimental conditions as the wild-type cells. VPA, one of the most studied HDAC inhibitors, was also applied to the knockdown cells as a positive control. Our results showed that neither resveratrol nor curcumin could rescue the neurite outgrowth defect of the SMN knockdown cells. However, VPA completely restored the defect and increased the neurite length significantly in both the knockdown and wild-type cells. van Bergeijk et al. previously reported that VPA promoted the neurite outgrowth of wild-type PC12 cells in a SMN-independent manner [43]. We also showed that the SMN protein level is not important for VPA-induced neurite elongation. On the other hand, even though curcumin failed to rescue the outgrowth defect, it lost its neurite-promoting ability when the SMN level was decreased. We investigated whether the neurite elongation was caused by increasing the SMN protein level in the wild-type cells. The number of nuclear bodies and total SMN protein level were investigated after the curcumin treatment. We found that the nuclear body numbers and total SMN protein level remained unchanged; however, previous studies showed that curcumin increased the SMN protein expression in patient fibroblast cells [33, 34]. One of the reasons for the differential action of curcumin may be due to the* SMN2* gene, which is present in humans, but not in rat genomes. Although PC12 cells have no* SMN2* gene, VPA treatment is capable of upregulating the SMN protein in this cell line, indicating that VPA and curcumin may act on different molecular targets [43]. Moreover, according to the literature, the total SMN protein level increases during differentiation, which was not observed in the present study, probably due to variations in the experimental conditions [19]. On the other hand, neurite outgrowth depends on the coordinated work of actin and microtubules to establish cytoskeletal networks and neurite morphology. Previous reports showed that curcumin affects the microtubule dynamics and SMN plays a role in the actin dynamics by affecting actin-binding/regulating proteins [30, 52]. Low levels of SMN protein may disrupt the cytoskeletal protein interactions and regulating pathways, which could be a possible explanation of why, in our study, the SMN knockdown cells were unresponsive to curcumin but the wild-type PC12 cells were not. Taken together, our results indicated that the neurite-promoting effect of curcumin was not caused by the SMN upregulation but was dependent on the SMN protein.

## 5. Conclusion

Our study was the first effort aimed at rescuing the neurite outgrowth defect using resveratrol and curcumin, but neither was found capable. However, our results indicated that curcumin needs the presence of the SMN protein to exert neurite-promoting activity. Using primary neurons or induced pluripotent stem cell-derived patient motor neurons will be valuable to increase the significance of our results. Further studies unveiling the detailed explanation of the function of the SMN protein and its complexes on neurites will help to improve our understanding of the outgrowth mechanism.
